# Mr. Wadley, on the Prevailing Epidemic

**Published:** 1803-06-01

**Authors:** T. W. Wadley

**Affiliations:** Surgeon. Stow on the Wold, Gloucestershire


THE request inserted in your last Number, induces me to send the following account of the late prevailing Epidemic, as it appeared in this neighbourhood, confining myself, however, to a relation of its symptoms and its general consequences. It may perhaps be necessary to premise, that the situation of the town is very high, and that the complaint has been particularly prevalent in it, and the neighbouring villages, (especially those in damp situations, and heretofore extremely subject to typhus mitior and scarlatina) though not entirely free from its effects, yet in comparison to the afflicted in the larger towns, may be said to have escaped.

The complaint, which was here likewise termed Influenza, began with symptoms of catarrhal fever, and first made its unwelcome appearance early in the month of March; and no malady has been more perplexing to the practitioner, nor the contra-indications so various, as the one I am about to describe. For the first week, symptoms appeared mild; those attacked, complained of languor and chilliness; the head (as they expressed it) seemed stuffed up, and a sneezing, accompanied with a sharp defluxion from the nose and eyes, took place. This appeared to me no other than the common effects of coryza, but the troublesome symptom was a dry, hard cough; yet a few were relieved by a pretty fair expectoration from the beginning, while others complained of soreness of the chest, with acute pains in the side. Nausea generally attended, and in some vomiting took place, but without any sensible relief; the head was greatly affected from the first attack, and great prostration of strength was a never-failing attendant; in a very few, delirium supervened. A state of costiveness generally prevailed, and from the beginning the urine was turbid and of a reddish colour. These symptoms increased for a few days, when a free expectoration came on, the cough began to give way, and the urine to resume its natural appearance. In these mild cases, an emetic was premised, and afterwards a bolus composed of pulv. antimon. and pulv. rhei, in doses suited to the habit, but perspiration was easily excited; the cough was afterwards treated with expectorants; the lac ammon. with the tinctura or acet. scillæ was found beneficial; cial.; and after the bowels had been sufficiently open, a gentle anodyne was always serviceable. The pulse was universally weak, small, and frequent.

After the lapse of a few days the disease began to assume a more alarming and Proteus form; patients were attacked with violent pains in the back and head, particularly over the eyes, and complained of great soreness of the limbs, universal languor, and were extremely dispirited. The pulse was accelerated in all, but in some small and thready, (if I may use the term) and in others more full and oppressed. The stomach was always pointed to as sore, and having a load upon it. A dry, hard cough, with a sound as proceeding from a metal tube; and some had a constant rattling in the throat, with difficulty of breathing, like phlegm stopping up the avenues of life. Debilitating sweats accompanied the disease throughout, and were present in every instance. The tongue at first was white, but after a day or two became brown, and in many a black fur covered it. When the cough had continued some days, a most distressing raucedo took place in some, and a few were afflicted with an additional cause for commiseration, a long continued aphonia. The mode adopted for relief was as follows.

First, the exhibition of an emetic of pulv. ipecac. was always premised, which seldom failed of evacuating the stomach of a dark coloured, greenish, and most offensive fluid. Aperients were always rejected when given before an emetic, and an enema was found of no service. The pain in the head was constantly lessened and frequently removed by the vomit, and a freer expectoration sometimes relieved the cough. When costiveness was a very urgent symptom, and appeared to increase the heat and fever, a draught of pulv. jalapii and kali tartar. was given, which never failed of being followed by stools of a peculiar fœtor and black colour, and this state of the alvine discharge often accompanied the disease throughout. But evacuations of this kind were required sparingly after the commencement of the disease. Extreme lassitude and debility were the early and peculiar diagnostics of this unwelcome visiter, and a very gentle cathartic has in more than one instance occasioned syncope, and a slow return to convalescence. Had not the cough and state of the pulse, attended with other catarrhal symptoms, accompanied these, I should have deemed it a different disorder, so various and perplexing were the symptoms. Venæsection was very generally inadmissible; in short, out of a great great number of cases, I do not recollect but two where it was decidedly indicated. The effects of this disease were, a long continued debility, great tendency to relapse, and loss of taste remained long after every other symptom had vanished, even after a return of appetite; and some complained of a saltish taste during their illness. The majority of those in a convalescent state received benefit from the following. ℞. Infus. colomb. ten. ℥vj. natron prep. ʒjß. tinct. colomb. ʒij. M. f. infus.; of which a small wine glass full was taken three times a day. Broths and oily medicines always disagreed with the few they were allowed to on the first appearance of the disease, before its nature was better known; and the sweats were relieved in many by the use of the acid. muriat. in common drink; oranges were eagerly coveted by the sick, and freely indulged in.

From observations made during the progress of the disease, I am inclined to believe it contagious from atmospheric influence, (and originating from that cause) and not from animal infection, though it generally affected a whole family where an individual of it fell ill. Though in the beginning a cautious depleting practice might have been judiciously employed, the sequel convinced me of the alarming tendency of this Proteus disease. How Nosologists will class this Epidemic, I cannot say; but many symptoms of it may be found in the Coryza catarrhalis of Sauv. sp. 1; though, perhaps, the Catarrhus a contagio of Cullen, or Epidemicus, sp. 3. of Sauvages may be most proper.

I am very conscious of the imperfect manner in which I have drawn up these observations; but a compliance with your request was the motive for my sending them, and not the *cacoethes scribendi.*

*Stow on the Wold, Gloucestershire,* I am, &c.

*April* 16, 1803. T. W. WADLEY, Surgeon.

P. S. Blisters to the stomach were very useful.

---

